# A novel *COMP* mutation in a Chinese family with multiple epiphyseal dysplasia

**DOI:** 10.1186/s12881-020-01040-y

**Published:** 2020-05-27

**Authors:** Jiashen Shao, Sen Zhao, Zihui Yan, Lianlei Wang, Yuanqiang Zhang, Mao Lin, Chenxi Yu, Shengru Wang, Yuchen Niu, Xiaoxin Li, Guixing Qiu, Jianguo Zhang, Deciphering Disorders Involving Scoliosis and COmorbidities (DISCO) study, Zhihong Wu, Nan Wu

**Affiliations:** 1 Department of Orthopedic Surgery, Peking Union Medical College Hospital, Peking Union Medical College and Chinese Academy of Medical Sciences, No. 1 Shuaifuyuan, Beijing, 100730 China; 2 Beijing Key Laboratory for Genetic Research of Skeletal Deformity, No. 1 Shuaifuyuan, Beijing, 100730 China; 3 Graduate School of Peking Union Medical College, Beijing, 100005 China; 4 Key Laboratory of Big Data for Spinal Deformities, Chinese Academy of Medical Sciences, No. 1 Shuaifuyuan, Beijing, 100730 China; 5 Department of Medical Research Center, Peking Union Medical College Hospital, Peking Union Medical College and Chinese Academy of Medical Sciences, No. 1 Shuaifuyuan, Beijing, 100730 China

**Keywords:** Cartilage oligomeric matrix protein (COMP), Multiple epiphyseal dysplasia, Femoral head necrosis, Whole-exome sequencing

## Abstract

**Background:**

Multiple epiphyseal dysplasia (MED) is a skeletal disorder characterized by delayed and irregular ossification of the epiphyses and early-onset osteoarthritis. At least 66% of the reported autosomal dominant MED (AD-MED) cases are caused by *COMP* mutations.

**Methods:**

We recruited a four-generation Chinese family with early-onset hip osteoarthritis, flatfoot, brachydactyly, and mild short stature. An assessment of the family history, detailed physical examinations, and radiographic evaluations were performed on the proband and other family members, followed by the performance of whole-exome sequencing (WES). The pathogenicity of the candidate mutation was also analyzed.

**Results:**

An AD-MED family with 10 affected members and 17 unaffected members was recruited. The main radiographic findings were symmetrical changes in the dysplastic acetabulum and femoral heads, irregular contours of the epiphyses, a shortened femoral neck, and flatfoot. Lower bone density was also observed in the ankle joints, wrist joints, and knees, as well as irregular vertebral end plates. In the proband, we identified the missense mutation c.1153G > T (p. Asp385Tyr), located in exon 11 of the *COMP* gene. This mutation was assessed as 'pathogenic' because of its low allele frequency and its high likelihood of co-segregation with disease in the reported family. Sanger sequencing validated the novel heterozygous mutation c.1153G > T (p. Asp385Tyr) in exon 11 of *COMP* in all affected individuals in the family.

**Conclusions:**

Our results underlined a key role of the Asp385 amino acid in the protein function of COMP and confirmed the pathogenicity of the *COMP* (c.1153G > T; p. Asp385Tyr) mutation in AD-MED disease. We have therefore expanded the known mutational spectrum of *COMP* and revealed new phenotypic information for AD-MED.

## Background

Multiple epiphyseal dysplasia (MED; MIM# 132400) is a skeletal disorder characterized by delayed and irregular ossification of the epiphyses as well as early-onset osteoarthritis [1]. To date, six genes have been associated with MED, including five genes that cause autosomal dominant MED (AD-MED; *COMP*, *COL9A1*, *COL9A2*, *COL9A3* and *MATN3*) and one gene that causes autosomal recessive MED (rMED; *SLC26A2*) [2–4]. The incidence of AD-MED is estimated to be 1 in 10,000 individuals, and at least 66% of reported AD-MED cases are caused by *COMP* mutations [5]. These cases are known as EDM1 (or *COMP*-MED), and are characterized by mild short stature, premature osteoarthritis of load-bearing joints, and abnormalities of the epiphyses of hands, long bones, and hips [6, 7].

The *COMP* gene encodes cartilage oligomeric matrix protein (COMP) made up of 757 amino acids [8]. COMP is a 552 kDa pentameric adhesive glycoprotein that is mainly found in synovium, tendons, ligaments, and the extracellular matrix of cartilage [9, 10]. The binding of COMP to extracellular matrix proteins is essential for the integrity of the cartilage and extracellular matrix [8]. Since the 1990s, more than 80 novel mutations involved in the pathogenesis of MED have been reported in *COMP* [9, 11]. The locations of these mutations are predominantly concentrated in the highly conserved type III (T3) calcium-binding repeat domain, and it has been demonstrated that the T3_4_ mutation is significantly associated with MED compared with other T3 repeats. These mutations affect the secretion of extracellular matrix proteins and extracellular matrix integrity, often leading to skeletal abnormalities including pseudoachondroplasia (PSACH) and MED [12].

Here, we report a four-generation Chinese family with early-onset hip osteoarthritis, flatfoot, brachydactyly, and mild short stature. An assessment of the family history, detailed physical examinations, and radiographic evaluations were performed on the proband and other family members, followed by the performance of whole-exome sequencing (WES). The pathogenicity of the candidate mutation was then analyzed.

## Methods

### Patients

The proband (III-10) was initially diagnosed with Legg–Calvé–Perthes disease based on the observed radiographic changes, including uneven density of the bilateral femoral head and bilateral femoral head collapse, which indicates avascular necrosis of the femoral head. After an investigation of the proband’s family history, a four-generation pedigree with 27 family members was recruited from Shanxi Province, China. Clinical evaluation including medical history, physical examination, and radiographic assessment, as well as peripheral venous blood (EDTA-K2 anticoagulant) was collected from all recruited individuals. For patients who cannot visit our hospital for clinical evaluation, we collected their clinical information by telephone interviews. Their consent forms and anteroposterior radiographs of pelvis were delivered by the express. Written informed consent was obtained from each participant; if the participant was younger than 16 years old, written informed consent was obtained from their parents or legal guardians. The Ethics Committee of Peking Union Medical College Hospital (PUMCH) approved this study.

### DNA preparation and WES

According to the manufacturer’s protocols, genomic DNA samples were extracted from peripheral blood leukocytes of each family member by peripheral blood DNA extraction kit (QIAamp DNA Blood Mini Kit; Qiagen, Germany). Purified DNA was qualified by Nanodrop2000 (Thermo Fisher Scientific, Waltham, MA, USA) and quantified by Qubit 3.0 using the dsDNA HS Assay Kit (Life Technologies, Carlsbad, CA, USA).

WES was performed on genome DNA of the proband (III-10) and individual II-6, II-9, III-6, III-18, and IV-12. The genomic DNA was broken into 180 to 280 base pair (bp) fragments by ultrasonoscope. Illumina paired-end libraries were prepared from DNA samples. The exome sequenced captured by the SureSelect Human All Exon V6 + UTR r2 core design (91 Mb, Agilent) were sequenced on an Illumina HiSeq 4000 platform (Illumina, San Diego, CA, USA). The raw sequencing data were analyzed through Genome Analysis Toolkit (GATK, Version 3.4.0). The error assessment, variant calling and annotation were performed through in-house developed Peking Union Medical College Hospital Pipeline (PUMP) [13].

The interpretation of variants was performed according to the American College of Medical genetics and Genomics (ACMG) guideline [14]. The variants were filtered through the following procedures: (1) the allele frequency of variants is required to be less than 1% or absent from databases like 1000 Genomes Project (The 1000 Genomes Project Consortium 2015) and Genome Aggregation Database (gnomAD) (http://gnomad-old.broadinstitute.org/). (2) Variants were filtered out when they were synonymous mutation or located in introns without influence on splicing and biological function. (3) Variants were reviewed by taken into consideration of phenotype evaluation, inheritance model, reported documents as well databases like Human Gene Mutation Database (HGMD) and Online Mendelian Inheritance in Man (OMIM) (available at: https://omim.org/).

### Sanger validation

A pair of PCR (Polymerase chain reaction) primers (F: 5′-CCATGAAGTTGGGACTCTGT-3′, R: 5′-GGTCATTTCTCTGGCAGTGT-3′) were designed using Primer 3 (http://primer3.ut.ee/) to amplify the Exon 11 of *COMP* gene. The PCR program was 95 °C for 3 min, followed by 38 cycles at 94 °C for 30 s, 58–60 °C for 30 s, 72 °C for 50 s, and a final extension at 72 °C for 8 min. All the collected samples in this family were used as DNA temple, the products of PCR analyzed in 2% agarose gels and purified by QIA quick PCR purification kit (Qiagen, Germantown, USA). Sequencing was performed on ABI3700 sequence Detection System (Applied Biosystems, Inc., Foster City, CA, USA). The reference sequence of the candidate gene was obtained from UCSC Genome Browser (http://genome.ucsc.edu) and compared with sequencing data through CodonCode Aligner (version 6.0.2.6; CondonCode, Centerville, MA, USA).

3-D protein structures of both wild and variant type COMP protein were predicted using an online modeling server, SWISS-MODEL program (https://swissmodel.expasy.org/), which were then viewed and edited by the molecular visualization system PyMOL (PyMOL Molecular Graphics System, Version 2.3.3, Schrödinger, LLC).

## Results

### Patients’ characteristics

**Fig. 1:**
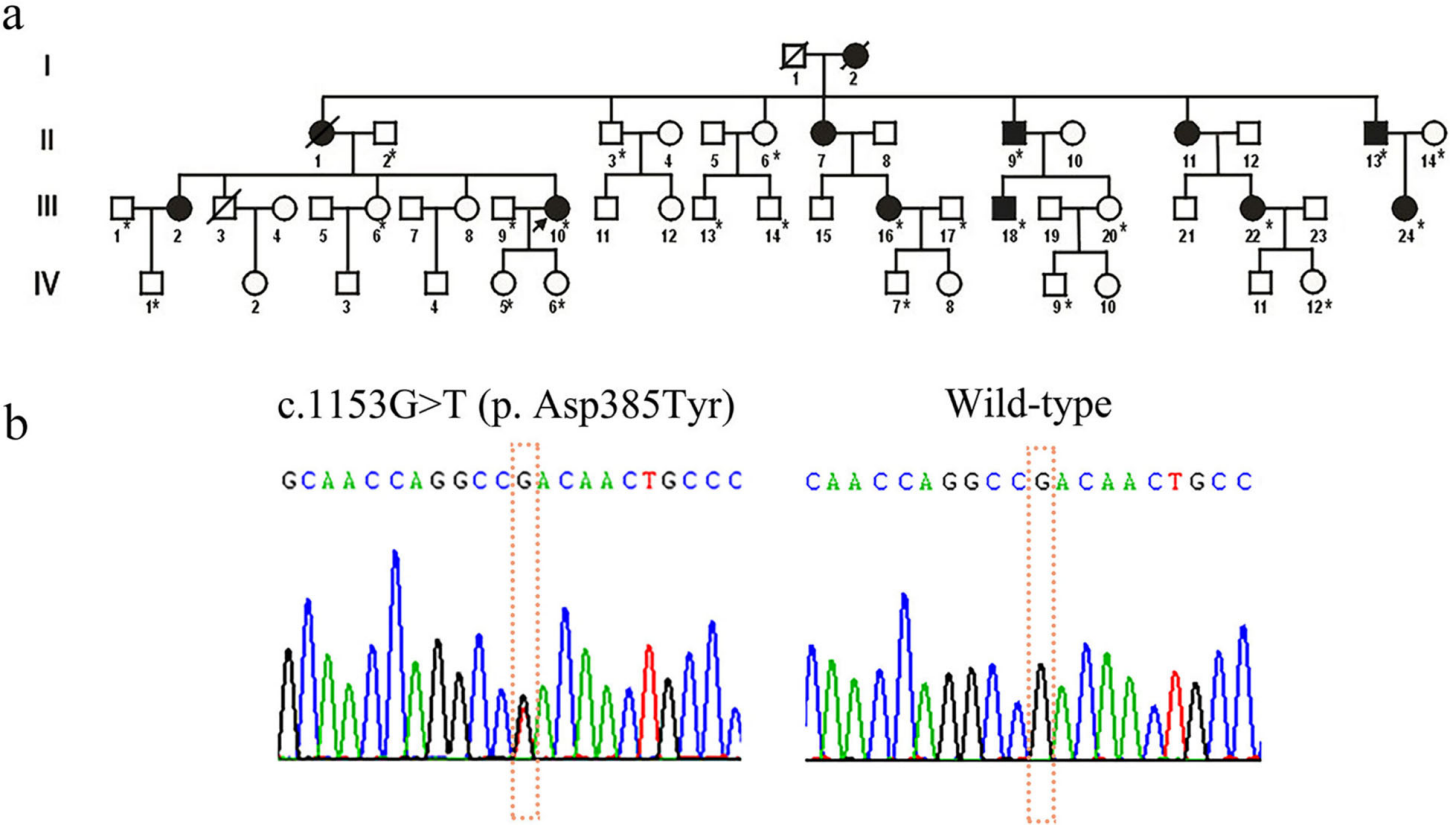
The pedigree of the Chinese family with MED and the result of Sanger sequencing. (**a**) *Family members on which DNA sequence analysis was performed; shaded symbols represent the affected individuals; black arrow represents the proband; slanting lines represent deceased individuals; (**b**) Electropherograms of Sanger sequencing showing the heterozygous c.1153G > T

**Fig. 2:**
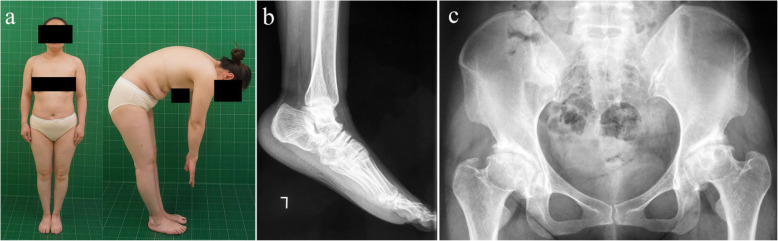
The clinical and radiological characteristics of the proband. Clinical and radiological appearance of proband (III-10) showing (**a**) limitation of movement in the lumbar spine, (**b**) flatfoot on both sides, and (**c**) the avascular necrosis of the bilateral femoral heads

**Fig. 3:**
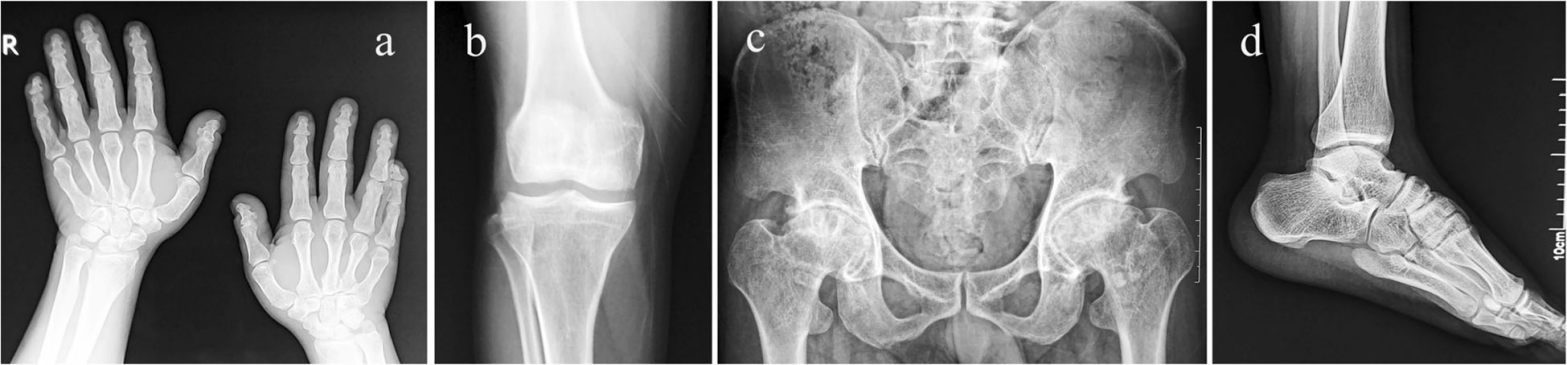
The radiological characteristics of an affected patient (II-13). Radiological assessment of a patient (II-13) showing (**a**) the brachydactyly of the both hands, (**b**) shallow femoral trochlear grooves and slightly squared femoral condyle in the knees, (**c**) the avascular necrosis of the bilateral femoral heads, and (**d**) flatfoot on both sides

We recruited an AD-MED family with 10 affected members and 17 unaffected members (Fig. 1a). The proband (III-10) was a 38-year-old woman who had developed a waddling gait at the age of 6 years (Fig. 2). At around the age of 22 years, the patient reported pain in the bilateral hip, lumbar vertebrae, and bilateral knees. The height of the proband was normal (152 cm), and there was no evidence suggesting growth retardation in her childhood. The main radiographic findings were symmetrical changes in the dysplastic acetabulum and femoral heads, irregular contours of the epiphyses, a shortened femoral neck, and flatfoot (Fig. 2). Decreased bone density of the ankle joints, wrist joints, and knees was observed, as well as irregular vertebral end plates. Her fingers and elbows were normal. When examining the other affected family members, we found one patient (III-2) with a mild phenotype (only presenting with the symptoms of hip pain), one patient (III-18) with mild sacroiliitis, and two patients with brachydactyly (II-9, II-13) (Fig. 3). All affected individuals in this family had flatfoot, except for one patient (III-2) (Table 1). Furthermore, compared with the general Chinese male population (average height = 170 cm), male patients in this family have generally shorter stature (≤165 cm).

**Table 1:** Clinical information of affected family members

Patient	Gender	Age at examination	Height	Flatfoot	Brachydactyly	Gait abnormalities (onset age)	Hip pain (onset age)	Other joint pain, bilateral or unilateral (onset age)	Morning stiffness (onset age)	Fatigue with long-distance walking (onset age)
II-9^a^	M	58	160	+	+	+ (9 y)	Left side (28 y)	Left knee (28 y)	Hip (−)	+ (−)
II-11	F	60	160	+	Data unavailable	+ (−)	Bilateral (38 y)	Lumbar vertebra (38 y)	Ankle, hip (40 y)	+ (−)
II-7	M	62	152	+	Data unavailable	+ (−)	–	Bilateral knees (56 y)	–	–
II-13^a^	M	54	155	+	+	+ (9 y)	Bilateral (−)	Bilateral knees (8 y)	Knees (26 y)	+ (8 y)
III-2	M	49	162	–	–	–	Bilateral (47 y)	–	–	–
III-10^a^	F	39	162	+	–	+ (6 y)	Left side (22 y) bilateral (25 y)	Lumbar vertebra, bilateral knees (25 y)	Ankle, knees (20 y)	+ (12 y)
III-16^a^	F	31	164	+	–	+ (12 y)	Bilateral (29 y)	Left knee (29 y)	Hip (29 y)	+ (12 y)
III-18^a^	M	25	165	+	–	+ (8 y)	Left side (13 y) bilateral (20 y)	Lumbar vertebra (18 y)	Hip (18 y)	+ (8 y)
III-22^a^	F	34	162	+	–	+ (10 y)	Bilateral (28 y)	–	Hip (30 y)	+ (10 y)
III-24^a^	F	11	140	+	–	+ (10 y)	–	–	–	–

^a^Family members on which DNA sequence analysis was performed

### Mutation analysis

**Fig. 4:**
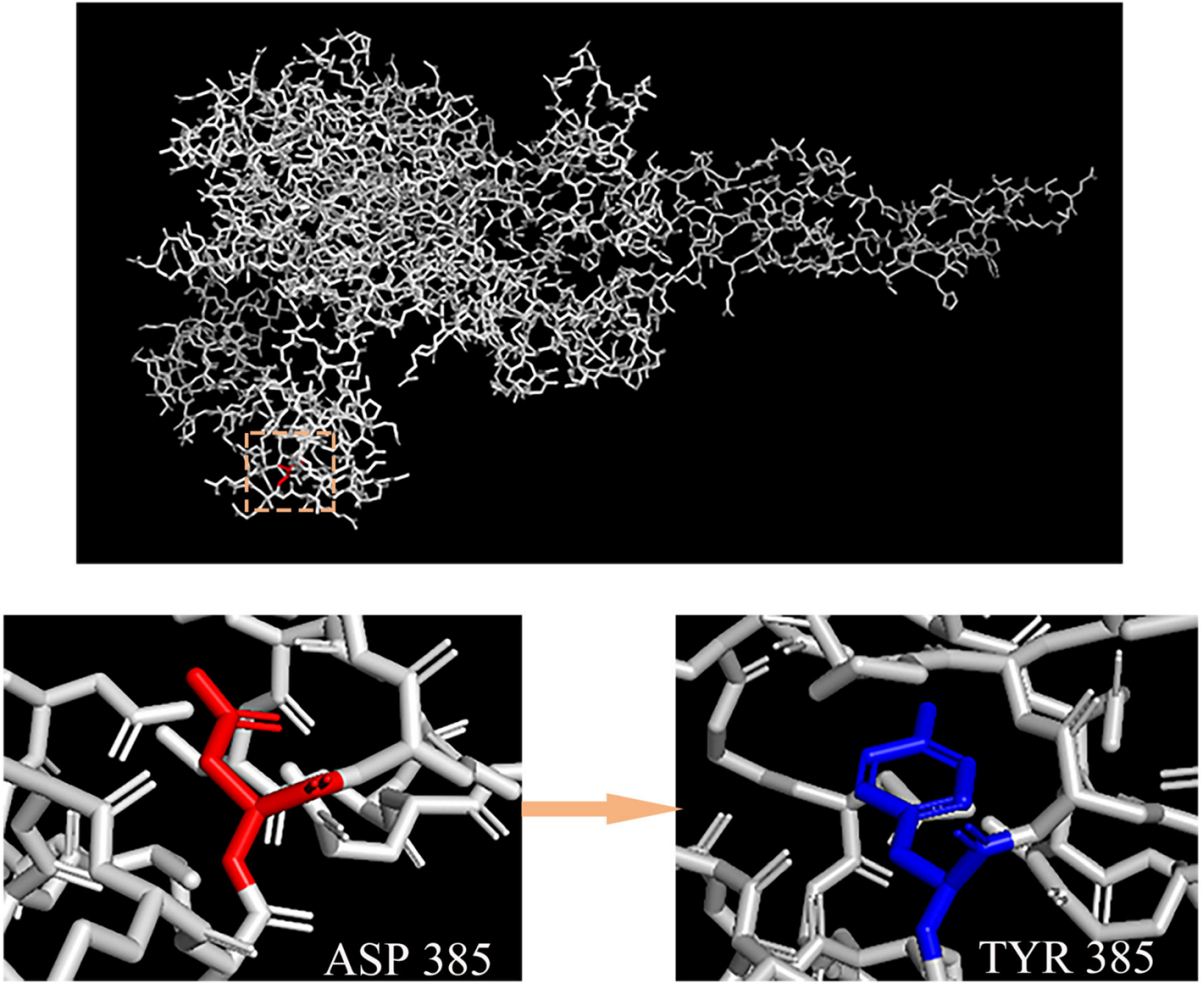
Protein structure predicted by SWISS-MODEL. Protein structure predicted by SWISS-MODEL shows replacement of the long side chain of Aspartate 385 by a phenolic hydroxy of tyrosine

After the raw sequencing data were processed through the analytical pipeline [13, 15], we identified that the individuals who underwent WES in total carried 3185 rare single nucleotide variants (SNVs), including missense, frameshift, splicing, and nonsense SNVs, as well as those with unknown influence, such as the synonymous or non-coding variants. All pathogenic and likely pathogenic variants were manually reviewed according to ACMG guidelines and the OMIM database. As a result, we identified the missense mutation, c.1153G > T (p. Asp385Tyr), located in exon 11 of *COMP*. This mutation was assessed as 'pathogenic' based on its low allele frequency and its high likelihood of co-segregation with disease in the reported family. Sanger sequencing was performed in 24 family members, including 7 affected members and 17 unaffected members (Fig. 1a). All affected family members carried the heterozygous mutation c.1153G > T (p. Asp385Tyr) in exon 11 of *COMP*, while the unaffected family members did not (Fig. 1b). These results further indicated that this mutation was co-segregated in our family and provides strong evidence for the pathogenicity of this mutation. The three-dimensional structure of the COMP protein provided further evidence of pathogenicity, with the mutation resulting in the replacement of the long side chain of Asp385 by a phenolic hydroxyl of tyrosine (Fig. 4).

In a previous study by Mabuchi et al., a different mutation at the same position (c.1153G > A, p. Asp385Asn) has been reported to cause MED [16]. Furthermore, Jackson et al. [17] also identified this recurrent mutation (c.1153G > A, p. Asp385Asn) in two British families and one Dutch family with MED. In addition, Liu et al. recently described an AD-MED family in which multiple members had been diagnosed with ANFH. Using WES, they identified a heterozygous variant in *COMP* (c.1153G > A) that contributed to the disease phenotype [7].

Consistent with the previously reported phenotypes, our pedigree cases also showed symptoms of classical AD-MED, such as femoral head necrosis, mild short stature, early-onset osteoarthritis of the knee and hip, and brachydactyly. These symptoms highlight the key role for Asp385 amino acid in the protein function of COMP and confirm the pathogenicity of *COMP* (c.1153G > T; p. Asp385Tyr) in AD-MED disease.

## Discussion

In the current study, we investigated a four-generation family with early-onset hip osteoarthritis caused by a heterozygous c.1153G > T mutation. Their prominent symptoms included severe osteoarthritis, knee pain, and femoral head necrosis, which is consistent with *COMP*-associated MED. According to the ACMG guideline and our study, this variation: a) is absent from the records of any frequency database, such as the 1000 Genomes Project and gnomAD, indicating that this mutation is rare (ACMG pathogenicity criteria: PM2); b) is located in a well-established functional domain (PM1); c) is co-segregated with disease in multiple affected family members (PP1); and d) has computational evidence supporting a deleterious effect on the gene (PP3). Based on these lines of evidence, we classified this mutation as pathogenic, with an important role in the genetic etiology of our family.

A different mutation at the same position (c.1153G > A, p. D385N) has been reported to cause MED [16]. In this previous study, the reported sporadic case had mild short stature and early onset osteoarthrosis, and was diagnosed with a severe form of MED—the “Fairbank type”. In another study, the authors reported a pedigree with severe hip osteoarthrosis. Using WES, a c.1153G > A mutation of the *COMP* gene was identified to be related to the MED phenotype of this family [17]. The phenotypes of the pedigrees reported by Liu et al. [7] were partially similar to that of our patients. They mainly manifested as necrosis of the femoral head, but their phenotype did not include flatfoot. In the current study, the c.1153G > T mutation was predicted to lead to an amino acid substitution from Asp to Tyr, implying that other substitutions at this position can also lead to the classical MED phenotype.

The precise function and pathogenic mechanisms of mutant COMP in MED have not been fully defined. However, compelling evidence indicates that COMP proteins play an important role in maintaining cartilage and extracellular matrix integrity. The misfolding of mutant COMP affects its normal secretion from the endoplasmic reticulum of chondrocytes, and this intracellular retention is toxic to these cells, resulting in premature chondrocyte death [8, 18]. These events reduce the number of chondrocytes in the growth plate, ultimately reducing linear growth, and the phenotypic outcome is dwarfism. Moreover, the reduction of COMP secretion also affects the assembly of collagen fibers [19], leading to a decrease in articular cartilage mechanical strength and the occurrence of early-onset osteoarthritis [12, 20]. In addition, the calcium-binding T3 repeat of COMP has been found to provide support for chondrocyte attachment [21]. Changes in the three-dimensional calcium-dependent structure of the mutant COMP may therefore alter chondrocyte attachment, thereby contributing to MED phenotype development. Briggs et al. [9] previously confirmed that both PSACH and MED-associated mutations are predominantly located within the T3 repeat domain of COMP (90% of mutations). They also reported that missense mutations and in-frame insertions/deletions of single residues in T3_5–7_ usually cause PSACH, while missense mutations in T3_3–4_ are more likely to cause MED. Our novel c.1153G > A mutation is located in the T3_3–4_ repeat of COMP. The aforementioned mechanisms may explain some of the phenotypes in our family, such as mild short stature and early-onset osteoarthritis.

Clubfoot is a rare radiological finding that has been observed mostly in association with rMED. In a previous study [22], rMED patients with clubfoot were reported to carry homozygous/compound heterozygous mutations in *SLC26A2* at birth. In addition, Superti-Furga et al. [23] described rMED patients with normal stature, clubfoot, and double-layered patellae caused by a *DTDST* mutation. In our study, all affected individuals in the family had flatfoot except for a single patient (III-2). However, other intra-familial differences in phenotype were observed in our family. For example, one patient (III-18) had mild sacroiliitis, with lower back pain at the age of 18, and also had gait abnormalities, mild short stature, flatfoot, and hip osteoarthritis. In addition, two male patients (II-9, II-13) had brachydactyly, but the other patients in this family did not have this phenotype. Furthermore, the male patients in this family were generally shorter in height, but it is unclear if this is related to the c.1153G > T mutation. In a pedigree study conducted by Sakamoto et al. [24], intra-familial differences in severity were also observed in their four-generation family: radiological manifestations in the knees were more severe in the proband’s father than in the proband, and the proband’s young sister was of shorter stature than the proband. Liu et al. [7] also reported intra-familial differences; for example, a twin brother in their reported family had more severe walking limitations than other family members. These intra-familial phenotypic differences may be difficult to explain by genetic factors but may be explained by the effects of environmental factors [1].

## Conclusions

We identified a novel heterozygous pathogenic mutation in *COMP* from an AD-MED family that exhibited *COMP*-associated MED, and other phenotypes including flatfoot. Our results expanded both the mutational and phenotypic spectra of *COMP* and suggested that this mutation of a key amino acid residue is disease-causing.

## Data Availability

The datasets generated during the current study are available in the Mendeley repository, 10.17632/gcfxv4yws3.1 (DOI: 10.17632/gcfxv4yws3.1). The datasets analysed during the current study include the variant frequency data from the gnomAD Browser repository; https://storage.googleapis.com/gnomad-public/release/2.1.1/vcf/exomes/gnomad.exomes.r2.1.1.sites.vcf.bgz; the variant frequency data from the 1000 Genomes Project repository: ftp://ftp.1000genomes.ebi.ac.uk/vol1/ftp/release/20130502/; the gene-disease relationship data from OMIM repository: https://www.omim.org/static/omim/data/mim2gene.txt; the hg19 reference genome from the UCSC Genome Browser repository: http://hgdownload.soe.ucsc.edu/goldenPath/hg19/bigZips/; the Genome Analysis Toolkit (GATK, Version 3.4.0) software: https://github.com/broadinstitute/gatk/releases/tag/4.1.7.0. The variant-disease relationship data from the Human Gene Mutation Database (HGMD) is not publicly available (the public version of this database is available only to registered users from academic institutions / non-profit organisations or commercial users who purchase a license of HGMD Professional), but can be required by contacting the corresponding author (Nan Wu) at dr.wunan@pumch.cn.

## Abbreviations

MED: Multiple epiphyseal dysplasia
AD-MED: Autosomal dominant MED
WES: Whole-exome sequencing
COMP: Cartilage oligomeric matrix protein
PSACH: Pseudoachondroplasia
PUMCH: Peking Union Medical College Hospital
ACMG: American College of Medical genetics and Genomics
OMIM: Online Mendelian Inheritance in Man
HGMD: Human Gene Mutation Database
PCR: Polymerase chain reaction
SNVs: Single nucleotide variants
rMED: Autosomal recessive MED
